# Prognostic value of the modified clot burden score in predicting outcomes of acute ischemic stroke patients

**DOI:** 10.1186/s12883-026-04626-w

**Published:** 2026-02-02

**Authors:** Kaveh Bahrami, Mohsen Soltani Sabi, Payam Sasannejad, Seyed Sajjad Alavi-Kakhki, Reza Nejad Shahrokh Abadi, Kavian Ghandehari

**Affiliations:** 1 Department of Neurology, School of Medicine, Mashhad University of Medical Sciences, Mashhad, Iran; 2 Department of Microbiology and Virology, Faculty of Medicine, Mashhad University of Medical Sciences, Mashhad, Iran

**Keywords:** Clot burden score, Stroke, Outcome, Prediction, Ischemic, Stroke large vessel occlusion, TOF-MRA, Stroke prognosis

## Abstract

**Background:**

Acute stroke is one of the leading causes of mortality and disability worldwide. In recent years, various scoring systems have been introduced to predict the outcomes of acute stroke patients, one of which is the Clot Burden Score (CBS). Therefore, the present study evaluates the prognostic value of the modified CBS in patients presenting with acute ischemic stroke.

**Methods:**

This study was conducted from 2022 to 2024 at Ghaem Hospital, Mashhad. Patients presenting to the hospital’s emergency department with symptoms of acute ischemic stroke were identified based on NIHSS clinical examination. NIHSS was assessed at admission, ASPECTS on baseline imaging, and the modified Rankin Scale at admission (mRS0) and 90 days post-stroke (mRS90). CBS, DWI-ASPECTS, mRS, and the modified CBS were measured for each patient. The collected data were analyzed using SPSS statistical software, and the prognostic value of both the original and modified CBS was assessed for predicting different outcomes, including mortality, disability, and severe disease.

**Results:**

A total of 130 patients were included (mean age 64.9 ± 14.1 years; 53.1% male). Compared to CBS, mCBS showed stronger correlations with NIHSS (ρ=–0.569, *p* < 0.0001) and mRS90 (ρ=–0.568, *p* < 0.0001). For disability prediction (mRS90 ≥ 2), the AUC of mCBS was 0.807 versus 0.735 for CBS (*p* = 0.0019). For severity (NIHSS ≥ 15), the AUC was 0.778 versus 0.720 (*p* = 0.0156). For mortality, AUCs were 0.708 and 0.681, respectively, with no significant difference (*p* = 0.3821).

**Conclusion:**

Based on the findings of this study, the modified CBS has greater prognostic value than the original CBS for predicting outcomes in acute stroke patients. If confirmed by future studies, the modified CBS may be considered a potential replacement for the conventional CBS scoring system.

**Supplementary Information:**

The online version contains supplementary material available at 10.1186/s12883-026-04626-w.

## Introduction

Acute ischemic stroke (AIS) remains a leading cause of mortality and long-term disability worldwide, requiring timely and effective management to improve patient outcomes [1]. Among the key determinants of clinical outcomes in AIS are the extent and location of thrombus and the success of reperfusion strategies [2], particularly in cases involving large vessel occlusion (LVO). LVOs, which commonly affect the intracranial carotid artery (ICA), middle cerebral artery (MCA), and basilar artery (BA), are associated with higher risks of severe neurological deficits, disability, and mortality compared to strokes involving smaller vessels [3, 4].

Blockages in the anterior cerebral circulation are among the most serious stroke-related events, often leading to significant neurological damage or even death [5]. Today’s stroke treatments focus heavily on reopening blocked arteries to minimize damage to vulnerable brain tissue and potentially reverse neurological impairments [6]. However, successful revascularization requires more than just clearing the primary blockage; it also depends on restoring blood flow to the smaller, downstream vessels. Several factors play a role in this process, including the type and location of the clot, the techniques used to remove it, the extent of the blockage, and the quality of the brain’s collateral blood supply. Larger, more proximal clots are particularly challenging to treat and are linked to poorer outcomes compared to smaller, more distal clots. For instance, patients with a hyperattenuated middle cerebral artery (MCA) sign—a marker of a larger, proximally located clot—often experience worse recovery and larger areas of brain damage. Similarly, blockages that involve both the internal carotid artery (ICA) and MCA are more difficult to reopen and show slower neurological improvement compared to blockages confined to the MCA alone [6–9].

The Clot Burden Score (CBS) is a 10-point scoring system based on CT angiography (CTA) designed to quantify thrombus extent in the anterior circulation. Lower CBS values indicate a more extensive thrombus burden, with scores ranging from 10 (absence of thrombus) to 0 (complete multisegment occlusion). While CBS has been correlated with clinical outcomes, including recanalization success, infarct size, and functional recovery, its predictive value for advanced interventions such as mechanical thrombectomy remains a subject of ongoing investigation [10]. Moreover, the original CBS may lack sufficient sensitivity for certain outcome measures, particularly in complex or severe cases of AIS.

Several studies have validated the CBS as a predictor of infarct volume, recanalization success, and functional outcome (e.g., Tan et al., 2009; Santos et al., 2016). However, none of these studies have specifically addressed the disproportionate impact of ICA occlusion on collateral failure and infarct expansion. This gap in the literature motivated our modification of the CBS to better reflect the clinical significance of ICA involvement. To address these limitations, modifications to the CBS scoring system have been proposed. A modified CBS seeks to incorporate additional thrombus-related parameters and provide a more nuanced assessment of clot characteristics. In particular, occlusion of the internal carotid artery (ICA) can compromise blood flow to extensive cortical and deep brain regions, leading to larger infarcts and poorer functional outcomes. Based on both clinical experience and published evidence, patients with ICA thrombus have consistently shown worse prognosis compared to more distal occlusions [11]. Therefore, in our modified CBS, we increased the weight assigned to ICA involvement to better capture its disproportionate impact on stroke severity and recovery. These modifications aim to enhance the score’s prognostic and prognostic utility, particularly for predicting critical outcomes such as mortality, disability, and disease severity.

Several studies have validated the Clot Burden Score (CBS) for outcome prediction in anterior circulation stroke (Tan et al., AJNR 2009; Fahed et al., Stroke 2018; Kargiotis et al., Ther Adv Neurol Disord 2022). However, none of these addressed the specific prognostic impact of internal carotid artery (ICA) involvement within the CBS framework. Given that ICA occlusion often represents a more extensive and hemodynamically critical lesion, our study introduces an ICA-weighted modification (mCBS) to better capture its disproportionate effect on outcome prediction.

In this study, we aimed to compare the prognostic performance of the modified CBS (mCBS) with the original CBS in patients with anterior circulation AIS. We assessed their correlations with established clinical and imaging measures (NIHSS, ASPECTS, and mRS) to determine whether mCBS offers added prognostic value in outcome prediction.

## Materials and methods

### Study design and patient cohort

This prospective observational study included all consecutive patients presenting with symptoms of acute ischemic stroke (AIS) between 2022 and 2024 at Qaem Hospital, Mashhad, Iran. Eligibility criteria were age ≥ 18 years, confirmed anterior-circulation large vessel occlusion (LVO), and presentation within 24 h of symptom onset. Patients who arrived within 4.5 h of onset were additionally screened for intravenous thrombolysis (tPA) eligibility according to guideline-based treatment windows, while those without evidence of LVO or presenting beyond 24 h were excluded.

Upon admission, patients were immediately assessed by the stroke team and transferred to the MRI unit. Imaging included simultaneous MRI and MRA, performed rapidly due to the availability of two 24-hour active MRI machines at the hospital. This approach eliminated the need for contrast injection or renal status assessment for CTA. In this study, magnetic resonance angiography (MRA) was employed instead of computed tomography angiography (CTA). This approach aligns with our centre’s standard acute ischemic stroke protocol, wherein patients routinely undergo initial MRI, with MRA performed concurrently during the same session. This protocol not only enhances efficiency by obviating the need for additional patient transfers but also eliminates the risks associated with iodinated contrast media and ionising radiation. Furthermore, MRA is widely accessible across numerous centres, offers excellent reproducibility, and facilitates non-invasive vascular follow-up monitoring for patients. Special attention was given to patients with severe symptoms and high NIHSS scores to evaluate the possibility of large core infarct, as well as those with strokes of unclear onset time (e.g., wake-up strokes).

Patient images were reviewed by three neurology specialists with over ten years of experience (two stroke fellows and one neurointervention and Interventional neuroimaging fellowship), and clinical decisions were made based on imaging findings. All raters were blinded to clinical data and outcomes during image interpretation, and final scores were determined by consensus. Clinical data were recorded by the on-call neurologist and maintained in a database for follow-up purposes. Clinical assessments included the NIHSS at admission, ASPECTS on baseline imaging, and the modified Rankin Scale at admission (mRS0) and at 90 days post-stroke (mRS90). For the analysis, only patients diagnosed with unilateral MCA territory stroke who completed the 90-day clinical follow-up were included. Of the 345 patients presenting with acute stroke symptoms, 22 did not complete the 90-day follow-up and were excluded from the study. The final cohort consisted of 130 patients with acute ischemic stroke involving the anterior circulation, specifically the middle cerebral artery (MCA) with or without internal carotid artery (ICA) occlusion. No patients with anterior cerebral artery (ACA) infarcts were included in the analysis.

This study was conducted in accordance with the ethical principles outlined in the Declaration of Helsinki. The study protocol, entitled “Diagnostic Value of Modified Clot Burden Score in Patients with Acute Stroke,” was reviewed and approved by the Ethics Committee of Mashhad University of Medical Sciences on December 22, 2022 (approval code: IR.MUMS.MEDICAL.REC.1401.555). Informed consent was obtained from all participants prior to their inclusion in the study.

### MRI and MRA protocol

MRI and MRA evaluations were performed immediately upon patient arrival at the emergency department using 1.5 T MRI scanners from Siemens Medical System (Erlangen, Germany). The imaging protocol focused on diffusion-weighted imaging (DWI) and ASPECTS for infarct assessment. MRI was conducted by a single expert MRI technician using a Siemens 1.5 T scanner. Axial 3-mm T1-weighted and proton density-fat-saturated sequences were used for imaging.

### MRA Protocol

MRA was conducted using a 1.5 T scanner (Magnetom Vision; Avanto; I-class). The acquisition parameters for the 3D Time-of-Flight (TOF) MRA sequence were set as follows: a repetition time/echo time (TR/TE) of 25 ms/7 ms, a flip angle of 25 degrees, and FOV of 180 mm for the read direction with a phase FOV of 100%. The slice thickness was 0.5 mm with a slice oversampling of 14.3%. The actual bandwidth was 100 Hz/pixel, resulting in a voxel size of 0.7×0.7×0.5 mm. The total acquisition time for the MR imaging scan was 4 minutes and 58 seconds.

Techniques such as CTA, CTP, and recanalization assessments were not part of this study, as the focus remained on MRI and MRA findings along with clinical evaluations.

### Imaging Analysis

#### Scores

The *Alberta Stroke Program Early CT Score (ASPECTS)* was calculated using diffusion-weighted imaging (DWI) sequences to assess the extent of ischemic infarction [12]. Clinical outcomes were evaluated using the National Institutes of Health Stroke Scale (NIHSS) at admission and the Modified Rankin Scale (mRS) at 90 days post-stroke [13, 14].

The *Modified Rankin Scale (mRS)* is a widely used tool for evaluating functional outcomes in stroke patients. It assesses the degree of disability or dependence in daily activities, ranging from 0 (no symptoms) to 6 (death). This scale provides a standardized measure of recovery and helps determine the effectiveness of interventions over time [15]. For this study, the mRS was used at 90 days post-stroke to evaluate long-term functional outcomes, providing critical insights into patient recovery and overall prognosis.

The *Clot Burden Score (CBS)* is a grading system designed to evaluate the extent of thrombus within the proximal anterior circulation. It is scored on a scale from 0 to 10, where a score of 10 indicates no thrombus is present, and a score of 0 reflects complete multisegment vessel occlusion. Points are deducted based on thrombus location: 2 points are subtracted for thrombus in each of the supraclinoid internal carotid arteries (ICAs), the proximal half of the middle cerebral artery (MCA) trunk, and the distal half of the MCA trunk. Additionally, 1 point is deducted for thrombus in the infraclinoid ICA, the anterior cerebral artery (ACA), and each affected M2 branch. The system accounts for both partially and completely occlusive thrombi, providing a standardized method for assessing clot burden in ischemic stroke patients [10].

**Fig. 1:**
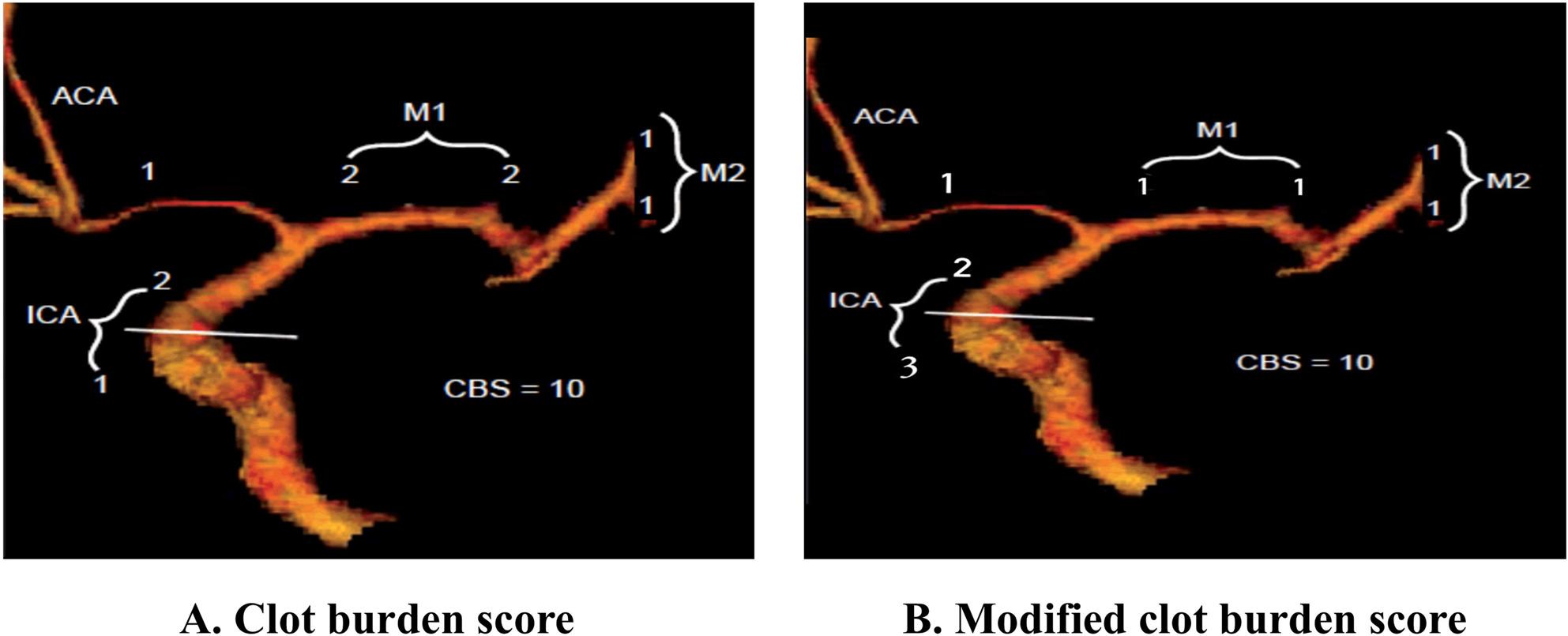
**A** Clot Burden Score illustrates the scoring system for large vessel occlusions with deductions based on thrombus location. A maximum score of 10 indicates no thrombus. **B** Modified Clot Burden Score refines the CBS by introducing additional scoring parameters, such as differentiation of thrombus characteristics and specific locations like the intracranial ICA, enhancing diagnostic precision. ICA = Internal Carotid Artery; ACA = Anterior Cerebral Artery; M1 = M1 Segment of the Middle Cerebral Artery; M2 = M2 Segment of the Middle Cerebral Artery

The *Modified Clot Burden Score (mCBS)* refines the standard Clot Burden Score (CBS) by incorporating additional parameters to provide a more detailed evaluation of thrombus extent and location within the anterior circulation. Like the CBS, the mCBS ranges from 0 to 10, with a score of 10 indicating no thrombus and 0 representing complete occlusion of multiple vessel segments. In the CBS (Fig. 1A), 2 points are subtracted for thrombus in the intracranial internal carotid artery (ICA), while in the mCBS (Fig. 1B), this deduction is increased to 3 points to reflect its critical importance. Additionally, in both systems, thrombus in the middle cerebral artery (MCA) trunk (proximal and distal halves) results in a deduction of 2 points each, while thrombus in the anterior cerebral artery (ACA) results in a deduction of 1 point. The mCBS further introduces more granular scoring by deducting 1 point for thrombus in each affected M2 branch. These refinements, seen in Fig. 1B, allow for a more precise assessment of thrombus burden and offer improved accuracy in predicting clinical outcomes and guiding therapeutic decisions.

All scoring, including the Clot Burden Score (CBS) and Modified Clot Burden Score (mCBS), was conducted based on the consensus of three neurology specialists, as described earlier. These specialists, including two stroke fellows and one neurointervention and neuroradiology fellow, carefully reviewed imaging findings to ensure accuracy and consistency in the evaluation of thrombus burden and its impact on clinical outcomes.

### Statistical analysis

All statistical analyses were conducted using the R software package (version 4.2.2). Results were presented as mean ± standard deviation (SD) or median (interquartile range [IQR]) for continuous variables and as proportions for categorical variables. The normality of the data was assessed using the Kolmogorov-Smirnov test, and non-normally distributed variables were analyzed using non-parametric methods. Baseline covariates including age, sex, and baseline NIHSS were examined for imbalance and did not show significant differences across groups, suggesting minimal confounding effect.

To assess correlations between variables, Spearman’s rank correlation test was applied. Patients were dichotomized based on the 90-day Modified Rankin Scale (mRS) score into good (mRS ≤ 2) and poor outcomes (mRS > 2). Patients with missing 90-day outcome data (*n* = 22) were excluded using a complete-case analysis approach. Group comparisons were performed using the Wilcoxon rank-sum test for continuous variables and Fisher’s exact test for categorical variables. Statistical significance was defined as a p-value < 0.05.

Logistic regression models were used to analyze the association between outcomes and variables of interest. Confounding factors such as age, sex, and treatment modality were included in multivariate models. Model adequacy was assessed using the Hosmer-Lemeshow test, while predictive performance was evaluated by the C-statistic. Receiver Operating Characteristic (ROC) curve analysis was conducted to calculate the Area Under the Curve (AUC) for CBS and modified CBS (mCBS) to evaluate their ability to predict mortality, disability, and stroke severity. All AUC values and correlation coefficients were reported with their corresponding 95% confidence intervals (CIs) to indicate the precision of the estimates.

The inter-rater reliability of CBS and mCBS was measured using the intraclass correlation coefficient (ICC), with values interpreted as poor (< 0.20), fair (0.21–0.40), moderate (0.41–0.60), good (0.61–0.80), and very good (0.81–1.0). These analyses provided a comprehensive statistical assessment of scoring systems and their relationship with clinical outcomes. A post-hoc power analysis was performed based on the observed AUC difference between CBS (AUC = 0.735) and mCBS (AUC = 0.807) for disability prediction, with a total sample size of 130 and α = 0.05. The calculated statistical power was 0.84, indicating adequate power to detect the observed effect.

## Results

### Demographic, scoring results, and outliers

**Fig. 2:**
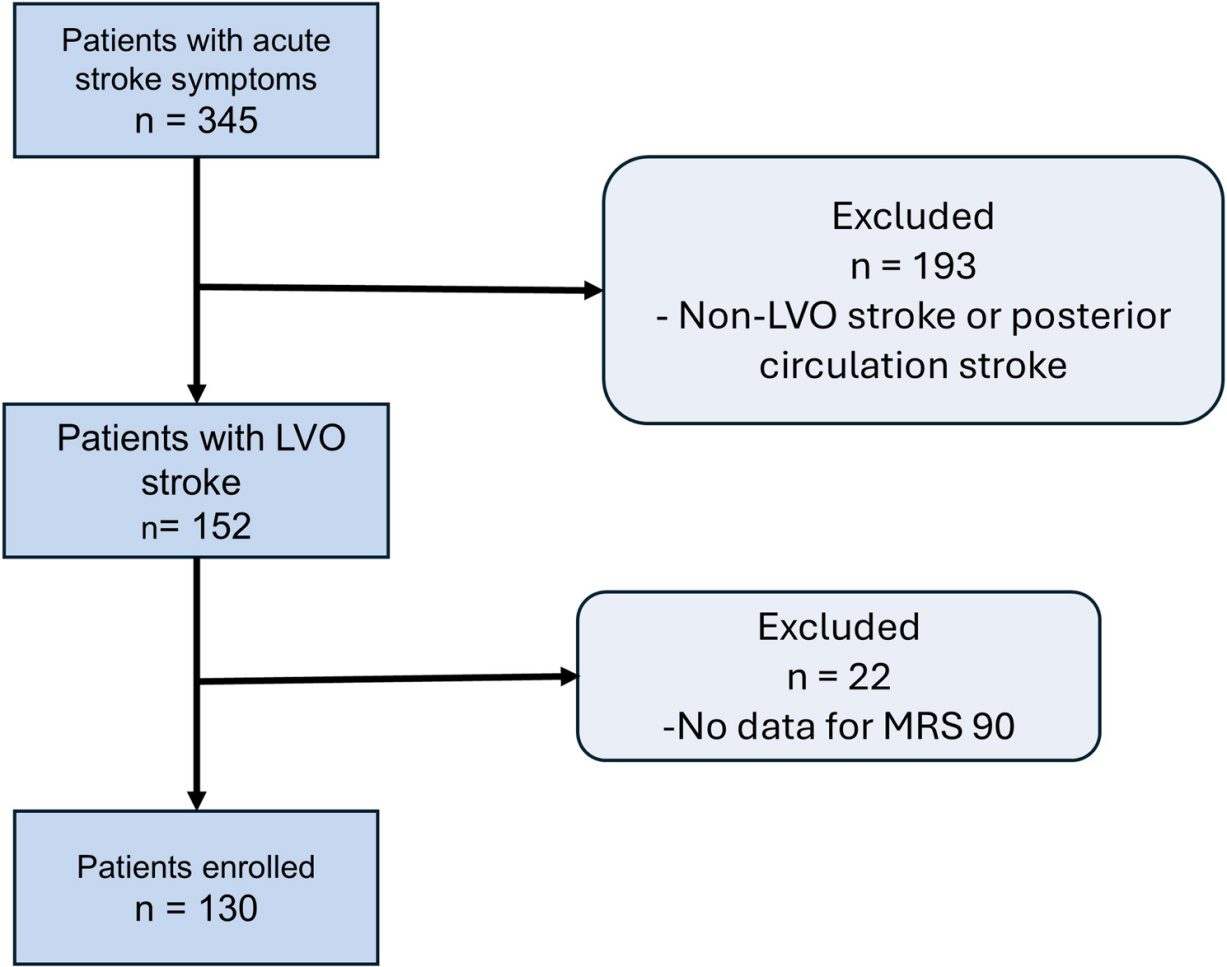
Flowchart shows patient selection. Among 345 patients with acute stroke symptoms, 193 are excluded due to non-large vessel occlusion (non-LVO) stroke or posterior circulation stroke. Of the remaining 152 large vessel occlusion (LVO) stroke patients, 22 are excluded for missing 90-day Modified Rankin Scale (mRS) scores, yielding a final cohort of 130 enrolled patients

A total of 130 patients with an average age of 64.85 ± 14.14 years (53.08% male) were included as seen in Fig. 2. All patients were evaluated using the NIHSS, ASPECTS, and mRS (at 0 and 90 days), along with CBS and modified CBS (mCBS). None of the scores followed a normal distribution, as confirmed by the Kolmogorov-Smirnov test. Notably, mCBS exhibited more potential outliers (22 cases) compared to other scoring systems. In addition, 22 patients from the original cohort did not complete the 90-day follow-up; the main reasons were relocation outside the catchment area (*n* = 10), inability to contact despite repeated attempts (*n* = 7), and withdrawal of consent (*n* = 5) (Table 1).

**Table 1:** Displaying the average values and inter-quartile range of the various scoring systems, along with distribution of normality and skewness of the data. Sym: Symmetrical, asym: Asymmetrical, IQR: Inter-Quartile range

Scoring System	Average ± SD	*P*-value	Interpretation	Skewness	Median	IQR [Q3-Q1]
NIHss	14.83 ± 3.74	0.0002	Not Normal	Sym	15	18 − 12
ASPECT	5.56 ± 2.28	< 0.0001	Not Normal	Asym Left	6	7 − 4
mRS 90	2.81 ± 2.3	< 0.0001	Not Normal	Sym	2	5 − 1
CBS	3.84 ± 2.48	< 0.0001	Not Normal	Sym	4	6 − 2
mCBS	4.82 ± 2.69	< 0.0001	Not Normal	Asym Left	6	6–3.75

### Correlations and significance

Prior to correlation analyses, inter-rater reliability between the two raters was assessed. Agreement was excellent, with ICC = 0.96 (95% CI: 0.94–0.98) for CBS and ICC = 0.97 (95% CI: 0.95–0.99) for mCBS. Spearman’s rank correlation revealed significant associations between CBS/mCBS and clinical scoring systems such as NIHSS, ASPECTS, and mRS. mCBS showed stronger correlations with these scores compared to CBS, particularly with disability (mRS90, rho = -0.5678, *p* < 0.0001) and stroke severity (NIHSS, rho = -0.5693, *p* < 0.0001). This highlights the enhanced predictive value of mCBS in stroke assessment (Table 2).

**Table 2:** Showing the results of the spearman’s rank correlation test against CBS and mCBS. CC: correlation coefficient

Scoring	Against CBS		Against mCBS	
	CC	*P*-value	CC	*P*-value
NIHss	-0.496	< 0.0001	-0.5693	< 0.0001
ASPECT	0.3618	0.00002	0.4058	< 0.0001
MRS 90	-0.4486	< 0.0001	-0.5678	< 0.0001

### Mortality, disability, and severity correlation

By the 90-day mark, 29 patients had died (mRS90 = 6), and 83 exhibited varying degrees of disability (mRS90 ≥ 2). Both CBS and mCBS correlated significantly with these outcomes, but mCBS consistently demonstrated stronger associations. For example, mCBS correlated with mortality (rho = -0.3126, *p* = 0.0002) and disability (rho = -0.532, *p* < 0.0001), indicating its potential as a superior tool for outcome prediction (Table 3).

**Table 3:** Correlation between CBS and mCBS and mortality, disability, and severity. Significant correlations using spearman’s rank test

Parameter	CBS		mCBS	
	Rho	*P*-value	Rho	*P*-value
Mortality	-0.2734	0.0016	-0.3126	0.0002
Disability	-0.411	< 0.0001	-0.532	< 0.0001
Severity	-0.399	< 0.0001	-0.5	< 0.0001

### ROC and AUC for mortality

**Fig. 3:**
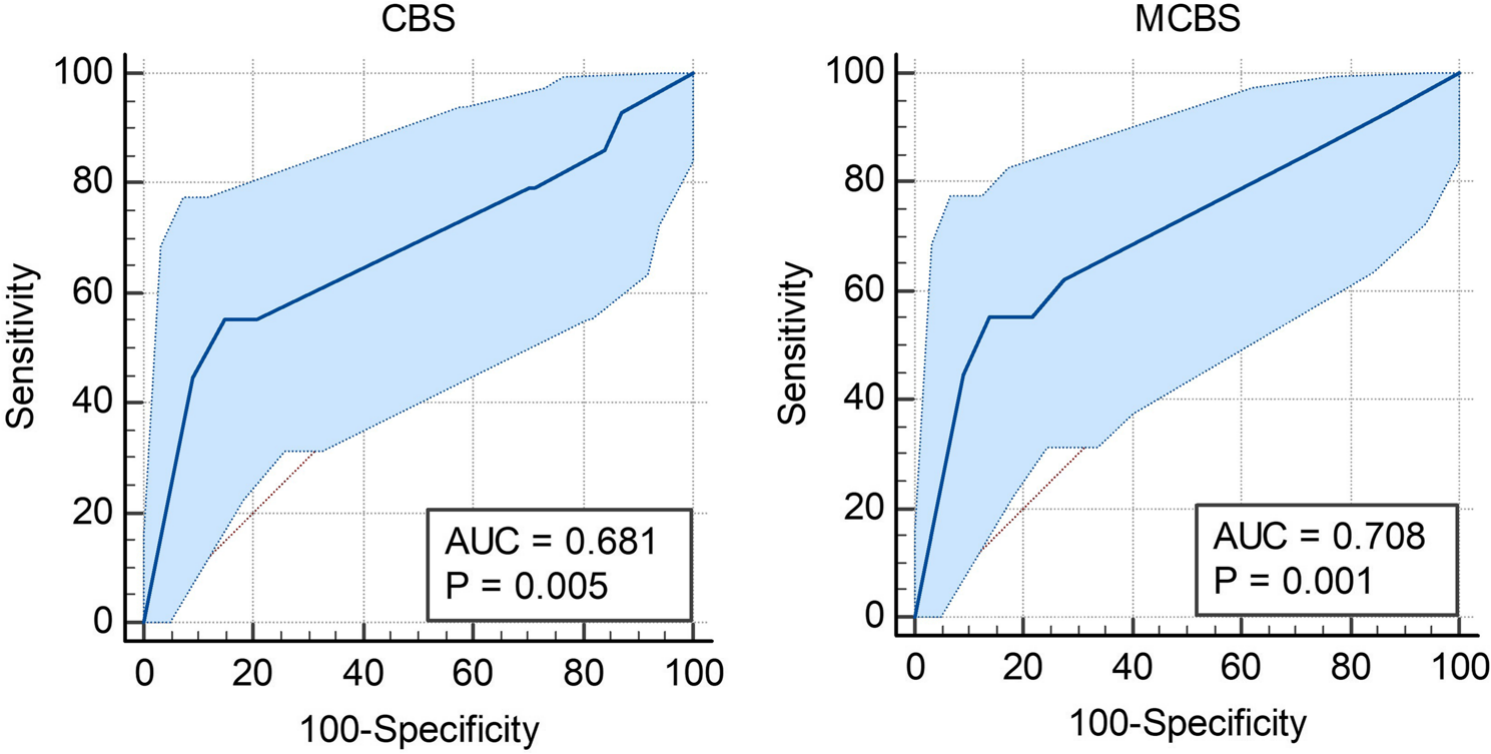
ROC curves show the performance of (**A**) CBS and (**B**) mCBS in predicting mortality outcomes with 95% Confidence Bounds. These are shown as the light blue shade around the plotted line

To further assess the predictive value of mCBS and CBS for mortality ROC curves and AUC were calculated (Fig. 3). For CBS, the AUC was calculated as 0.681 with a P-value of 0.0045 (95%CI [0.593, 0.760]). Additionally, CBS had a Youden index J of 0.4032, with the associated criterion “≤1” having a sensitivity and specificity of 55.17% and 85.15%, respectively. mCBS comparatively performed better, with an AUC of 0.708 and a P-value of 0.0005 (95%CI [0.622, 0.784]). However, the Youden index J (0.4131), sensitivity (55.17%), and specificity (86.14%) were nearly identical. However, pairwise comparison of ROC curves showed no statistical significance (P-value = 0.3821).

### ROC and AUC for disability

**Fig. 4:**
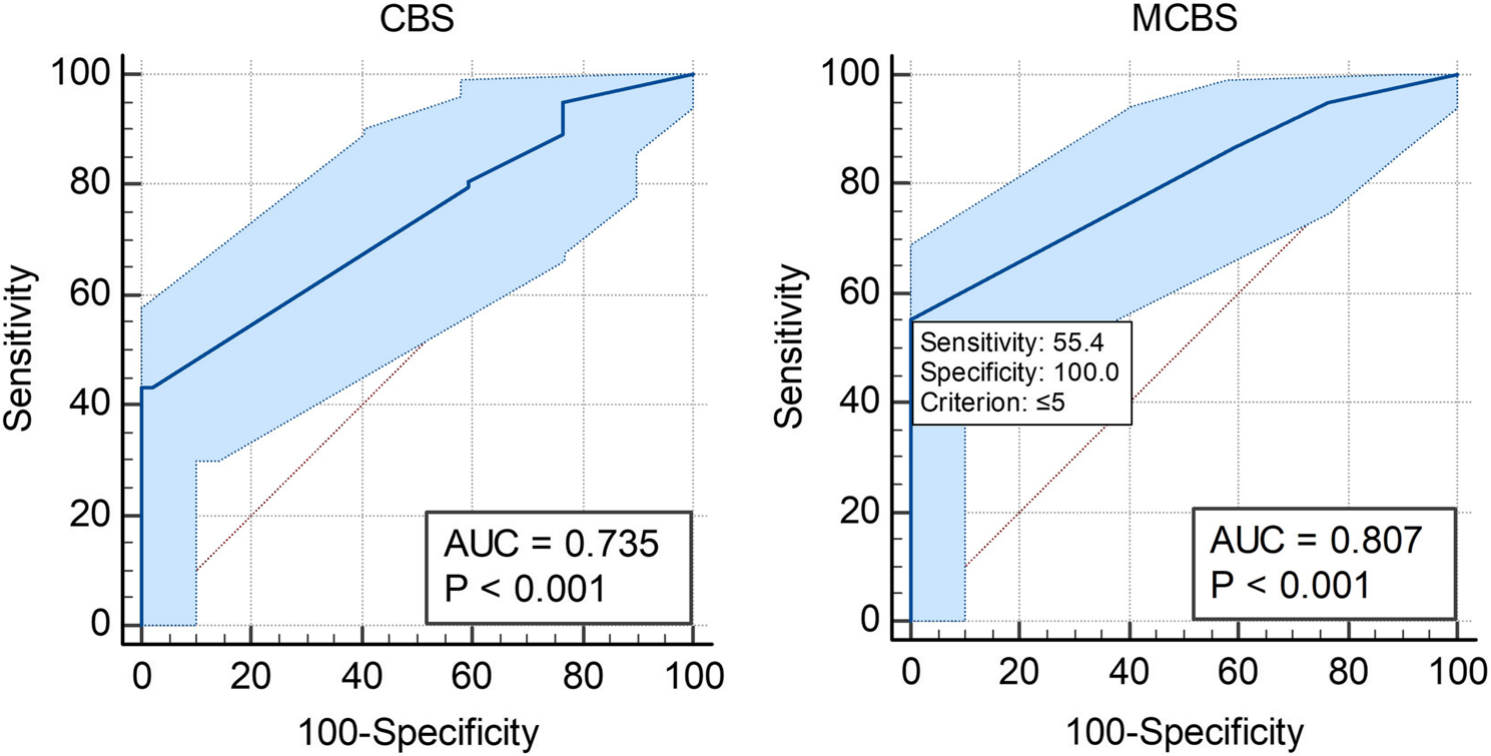
ROC curves show the performance of (**A**) CBS and (**B**) mCBS in predicting disability outcomes with 95% Confidence Bounds. These are shown as the light blue shade around the plotted line

To assess the predictive value of CBS and mCBS for disability the patients were categorized into two groups, those without disability at the 90-day mark (mRS90 0–1) and those with disabilities (mRS90 2–6). For CBS, the AUC was calculated as 0. 735 with a P-value of < 0.0001 (95%CI [0.651, 0.809]). The Youden J index was calculated at 0.4337, with the criterion “≤2” having a sensitivity and specificity of 43.37% and 100%. The AUC for mCBS was slightly higher at 0.807 (Fig. 4A ), with a similar P-value of < 0.0001 (95%CI [0.728, 0.871]). The J index was also higher at 0.5542 (Fig. 4B), however this time the criterion “≤5” had a specificity of 100% and a sensitivity of 55.42%. This time, unlike with mortality, pairwise comparison of the ROC curves was deemed statistically significant with a P-value 0.0019.

### ROC and AUC for severity

**Fig. 5:**
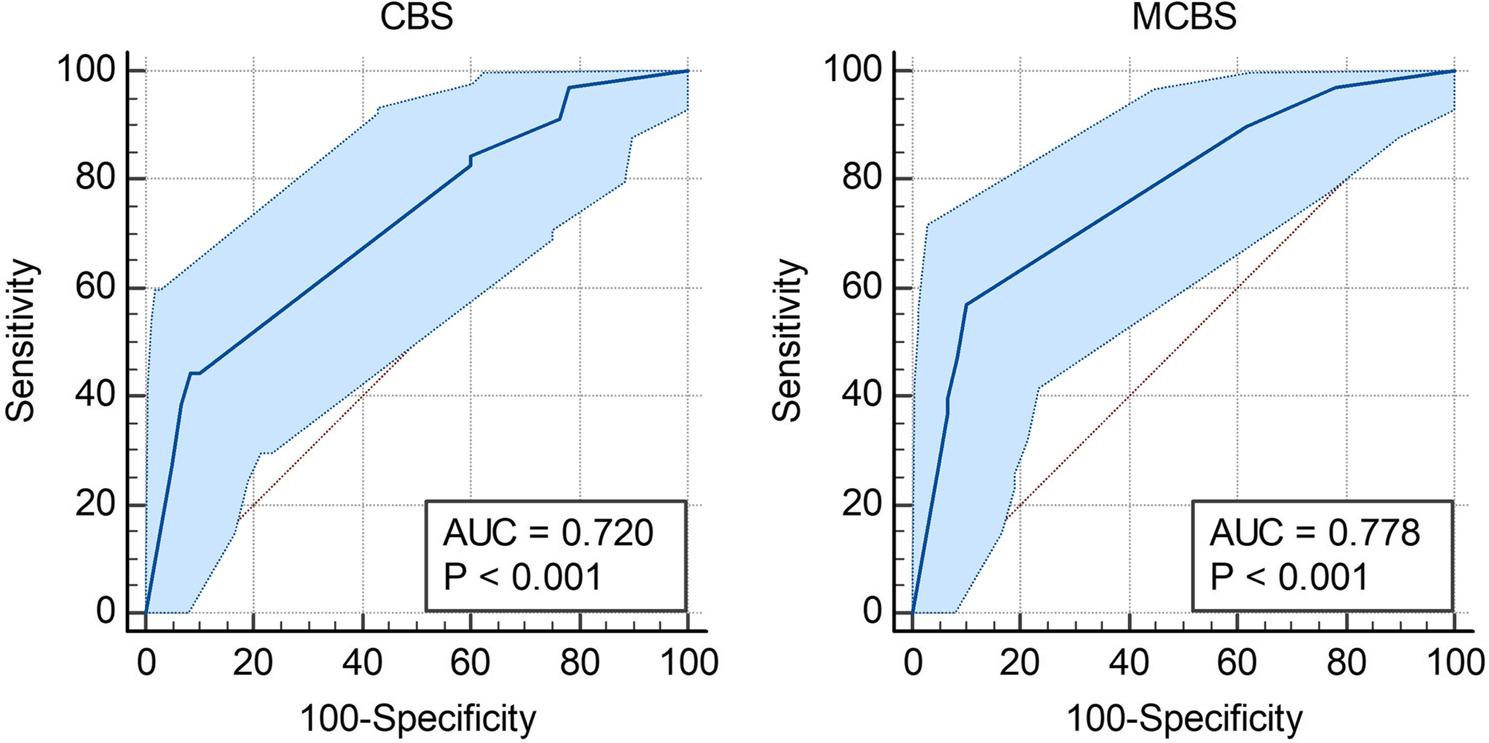
ROC curves show the performance of (**A**) CBS and (**B**) mCBS in predicting severity outcomes with 95% Confidence Bounds. These are shown as the light blue shade around the plotted line

To assess the predictive value of CBS and mCBS for severity the patients were categorized into two groups, severe stroke was rated as an NIHss score of 15 or higher. For CBS, the AUC was calculated as 0.72 with a P-value of < 0.0001 (95%CI [0.635, 0.795]). The Youden J index was calculated at 0.3595, with the criterion “≤2” having a sensitivity and specificity of 44.29% and 91.67%. The AUC for mCBS was slightly higher at 0.778 (Fig. 5A), with a similar P-value of < 0.0001 (95%CI [0.696, 0.846]). The J index was also higher at 0.4714 (Fig. 5B), however this time the criterion “≤5” had a specificity of 90% and a sensitivity of 57.14%. Similar to that of disability, pairwise comparison of the ROC curves was deemed statistically significant with a P-value 0.0156 (Table 4).

**Table 4:** Spearman’s rank test results

Parameter		mCBS		CBS	
		Rho	P-value	Rho	P-value
APT	Mortality	-0.387	0.0029	-0.369	0.0048
	Disability	-0.613	< 0.0001	-0.558	< 0.0001
	Severity	-0.387	0.0029	-0.378	0.0037
TPA	Mortality	-0.641	0.0023	-0.347	0.1335
	Disability	-0.662	0.0015	-0.470	0.0366
	Severity	-0.468	0.0373	-0.364	0.1143
EVT	Mortality	-0.0250	0.8587	-0.0524	0.7095
	Disability	-0.281	0.0414	-0.166	0.2356
	Severity	-0.510	0.0001	-0.405	0.0027

### Treatments and outcomes

**Fig. 6:**
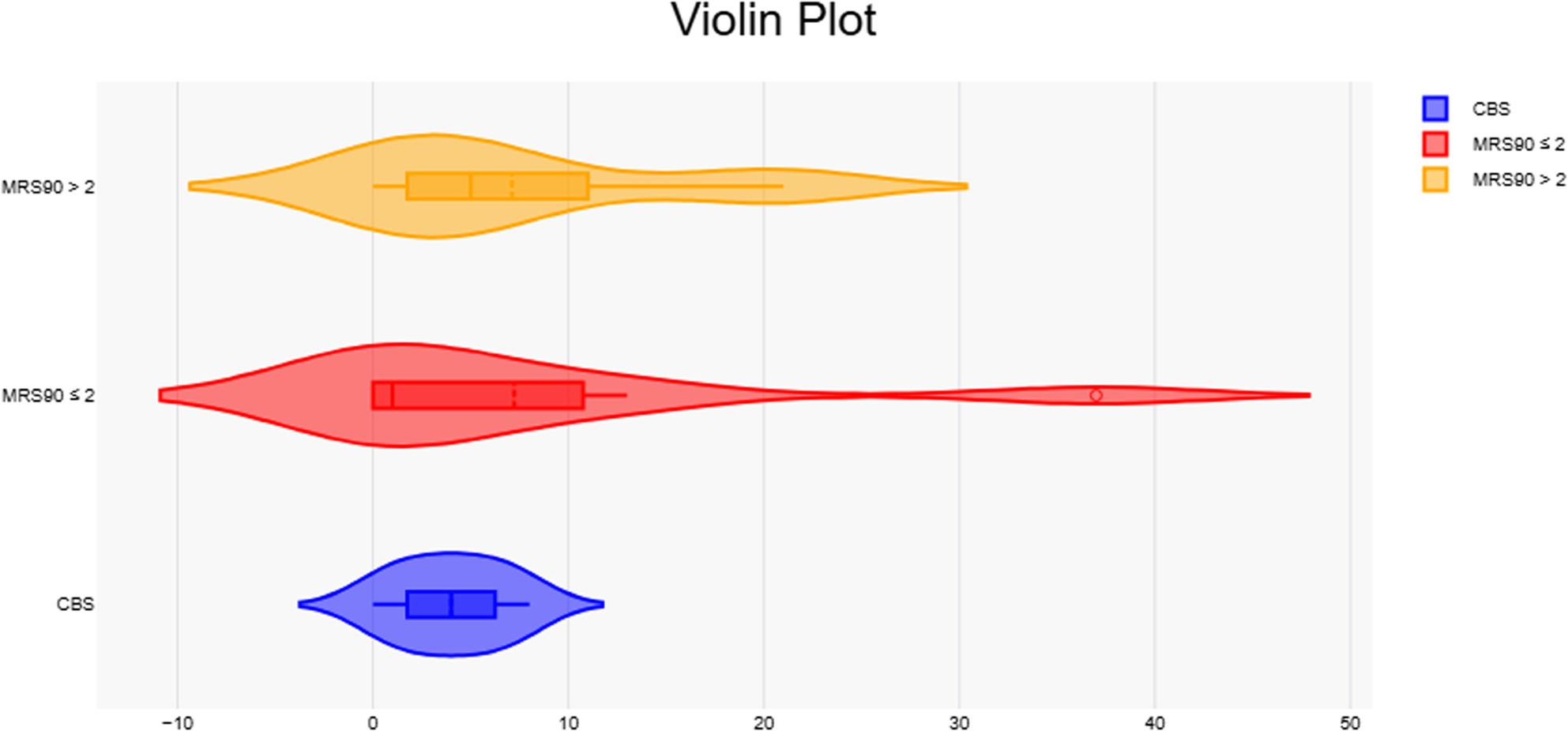
Violin plots showing the distribution of CBS values stratified by 90-day outcomes (mRS90 ≤2 vs. mRS90 >2). Visualization corresponds to patient counts and categories presented in Table 5

**Fig. 7:**
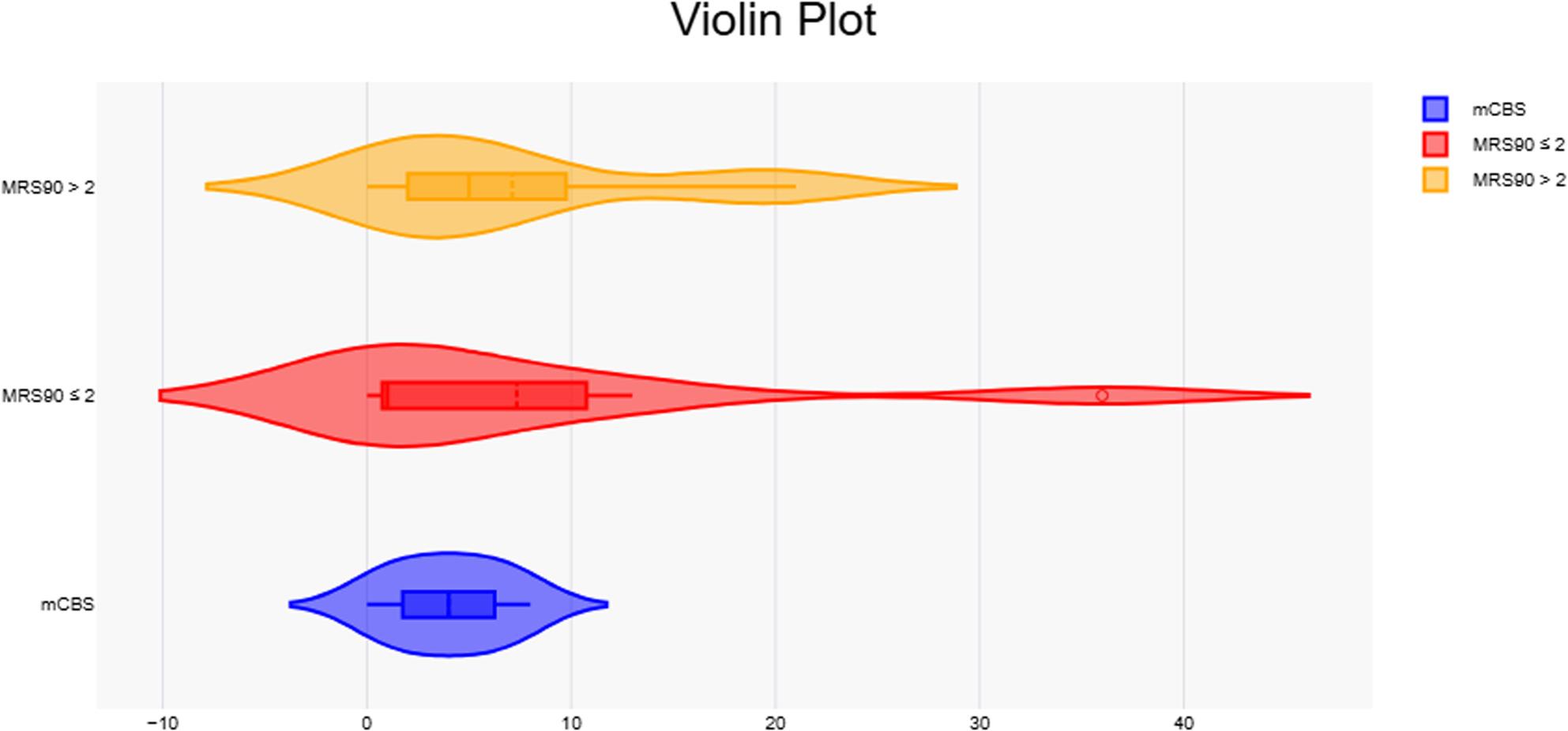
Violin plots showing the distribution of mCBS values stratified by 90-day outcomes (mRS90 ≤2 vs. mRS90 >2). Visualization corresponds to patient counts and categories presented in Table 5

In our study, 57 patients received APT^1^, 20 received TPA^2^, and 53 received EVT^3^. Differences in disability and severity were varied across all treatment modalities, although only patients that received APT had a significant reduction in mortality rates. MCBS was better correlated to outcomes within the different treatment groups, particularly with mortality and severity in those who received TPA, and disability in EVT patients (Figs. 6 and 7).

**Table 5:** Counts and categorization of patients according to mRS90 scores

CBS	MRS90 ≤ 2	MRS90 > 2	mCBS	MRS90 ≤ 2	MRS90 > 2
0	0	21	**0**	1	21
1	1	8	**1**	1	7
2	3	2	**2**	0	0
3	1	0	**3**	0	2
4	37	20	**4**	4	2
5	0	1	**5**	1	7
6	10	5	**6**	36	18
7	0	5	**7**	10	5
8	13	2	**8**	13	2

## Discussion

The modified Clot Burden Score (mCBS) represents a significant advancement in the evaluation of clot extent and its implications for clinical and radiologic outcomes in acute MCA territory strokes. Building upon the established utility of the Clot Burden Score (CBS), mCBS incorporates refinements that enhance its predictive accuracy and clinical applicability [10, 16].

Our findings align with previous studies indicating that smaller clot extents are associated with smaller baseline infarcts, lower baseline NIHSS scores, and more favorable clinical outcomes [10]. However, mCBS’s refined scoring system—which accounts for both proximal and distal vessel involvement—provides a more granular assessment of clot burden, enabling superior prediction of disability and severity outcomes compared to CBS. For example, mCBS demonstrated stronger correlations with NIHSS (ρ = -0.5693, *p* < 0.0001) and mRS90 (ρ = -0.5678, *p* < 0.0001) scores, underscoring its enhanced utility in prognostic assessment of acute stroke.

## Clinical and imaging integration

Stroke physicians often rely on clinical parameters such as baseline NIHSS scores and symptom onset timing when deciding on thrombolysis within the accepted treatment windows. Proximal clot location has emerged as a critical determinant of stroke outcomes, as larger clot burdens correlate with greater baseline infarction volumes and poorer clinical results. Scores like CBS account for clot location by assigning weighted points to terminal ICA and proximal MCA regions, while also considering distal MCA and ICA involvement. These refinements facilitate a nuanced understanding of clot extent and patient stratification [17].

Similar to CBS, mCBS is a reproducible and rapidly applicable tool that complements clinical parameters such as NIHSS and ASPECTS. While CBS studies have shown its utility as an independent predictor of radiologic outcomes, our results highlight the added prognostic value of mCBS in predicting both clinical and radiologic outcomes. Specifically, mCBS’s ability to incorporate detailed vessel location assessments—including proximal and distal MCA involvement—addresses limitations in traditional CBS metrics.

This enhanced performance is particularly evident in our study’s ROC curve analyses while the absolute AUC differences between CBS and mCBS were below 0.1, these changes—though modest—can be clinically meaningful when applied to individualized treatment selection and risk stratification in acute stroke settings. For instance, mCBS achieved an AUC of 0.708 for mortality prediction, surpassing CBS (AUC = 0.681), although the difference was not statistically significant. Therefore, the prognostic value of mCBS for mortality should be interpreted with caution and viewed as exploratory. Conversely, for disability and severity predictions, mCBS exhibited statistically significant improvements, reinforcing its role as a critical tool in patient stratification and treatment planning. While the AUC improvement between CBS and mCBS for mortality prediction was modest (0.027), even small gains in predictive accuracy can be clinically meaningful in acute stroke triage, especially when specificity reaches a very high level, as seen in disability prediction.

### Treatment implications

Mechanistically, ICA occlusion often results in compromised collateral circulation, leading to larger infarct cores and worse outcomes. This pathophysiologic rationale supports our weighting of ICA involvement in mCBS. When compared with prior CBS validation studies (Tan et al. 2009; Fahed et al. 2018; Kargiotis et al. 2022), our findings suggest that including ICA weighting provides a more physiologically representative assessment of thrombus impact.

Our findings suggest that mCBS provides a more nuanced framework for identifying patients who may benefit from aggressive recanalization strategies, particularly in centers where advanced imaging modalities are available.

Additionally, mCBS’s correlation with collateral flow parameters highlights its potential in assessing the interplay between clot extent, collateral supply, and final infarct size. While collateral supply plays a pivotal role in mitigating infarct progression until reperfusion is achieved, mCBS’s comprehensive vessel involvement scoring enhances the understanding of this interaction, providing valuable insights for personalized treatment approaches.

From a clinical perspective, the modified CBS can be feasibly applied in routine stroke imaging. The additional weighting for ICA involvement does not add significant complexity compared with the original CBS and can be performed rapidly by trained neuroradiologists. With advances in automated image analysis, mCBS could also be integrated into radiology workflows, allowing faster and more standardized prognostic assessment in acute stroke settings.

### Limitations and future directions

This study has several limitations. First, the findings were derived from a single-center cohort and have not been externally validated; therefore, generalizability to other populations and imaging protocols remains uncertain. Second, the subgroup of patients treated with intravenous tPA was relatively small, which may have limited the statistical power to detect treatment-specific associations. Third, clot burden was assessed using TOF-MRA rather than CTA or perfusion imaging, raising the possibility of misclassification in thrombus extent. This limitation is particularly relevant when comparing our results with CTA-based studies. Finally, posterior circulation and anterior cerebral artery (ACA) strokes were excluded, so the applicability of our modified CBS to these stroke subtypes cannot be determined. Accordingly, the generalizability of our findings beyond anterior circulation strokes remains limited. Future multicenter and external validation studies are needed to confirm the generalizability and reproducibility of mCBS across different imaging protocols and populations.

## Conclusion

In conclusion, mCBS builds upon the foundational work of CBS to offer a more detailed and clinically relevant assessment of clot burden in acute ischemic stroke. By integrating mCBS into standard clinical workflows alongside established imaging and clinical parameters, clinicians can achieve a more nuanced understanding of stroke pathophysiology, pending confirmation in future multicenter validation studies.

## Supplementary Information

Supplementary Material 1.

Supplementary Material 2.

## Data Availability

The datasets generated and analyzed during the current study are available from the corresponding author upon reasonable request.

## Footnotes

1 Antiplatelet therapy.

2 Intravenous tissue plasminogen activator.

3 Endovascular therapy.
