# Supplementary material for: Prognostic value of the modified clot burden score in predicting outcomes of acute ischemic stroke patients
Source: BMC Neurol. 2026 Feb 2;26:139. doi: 10.1186/s12883-026-04626-w (PMC12955203; doi:10.1186/s12883-026-04626-w)
Supplement: Supplementary file 1 — Supplementary Material 1. [file 12883_2026_4626_MOESM1_ESM.pdf]

3. Results

3-1. Demographic, Scoring Results, and Outliers

In this study a total of 130 patients were included, with an average age of 64.85±14.14 years, of which 69 (53.08%) were male. All patients were scored according to the NIHss, ASPECT, and MRS (at 0 and 90 days) criterias, in addition to the CBS and modified CBS scoring systems (Graph 1 and 2). There was no significant difference between the two genders (P-value 0.48). The results of the Kolmogorov-Smirnov Test showed that none of the scores were normally distributed (Table 1). According to the Tukey's fences test, only MRS0 and the modified CBS had any potential outliers, 2 and 22 respectively.

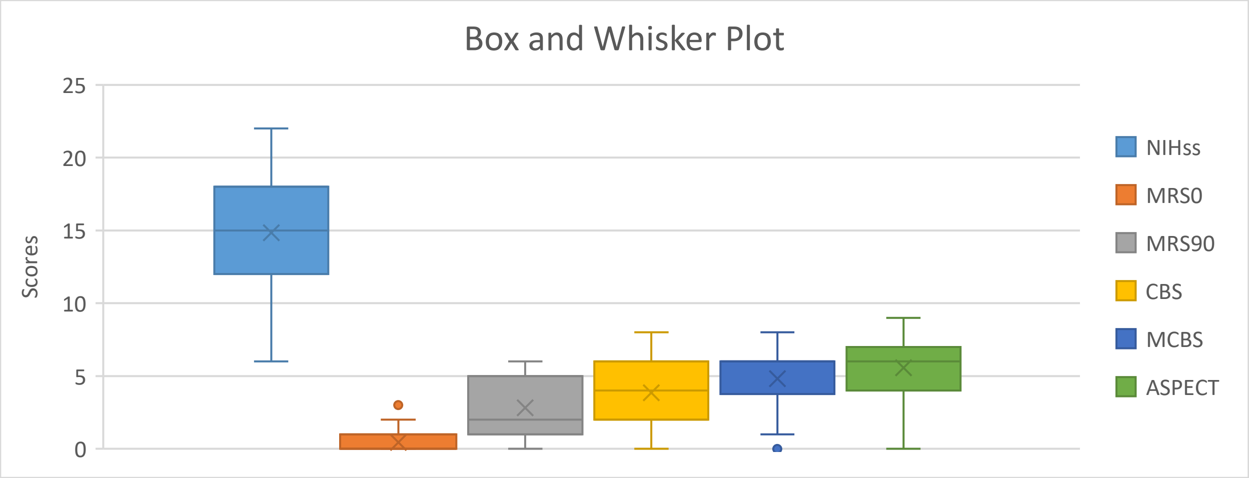

Chart 1. Box and Whisker Plot of the various scoring systems.

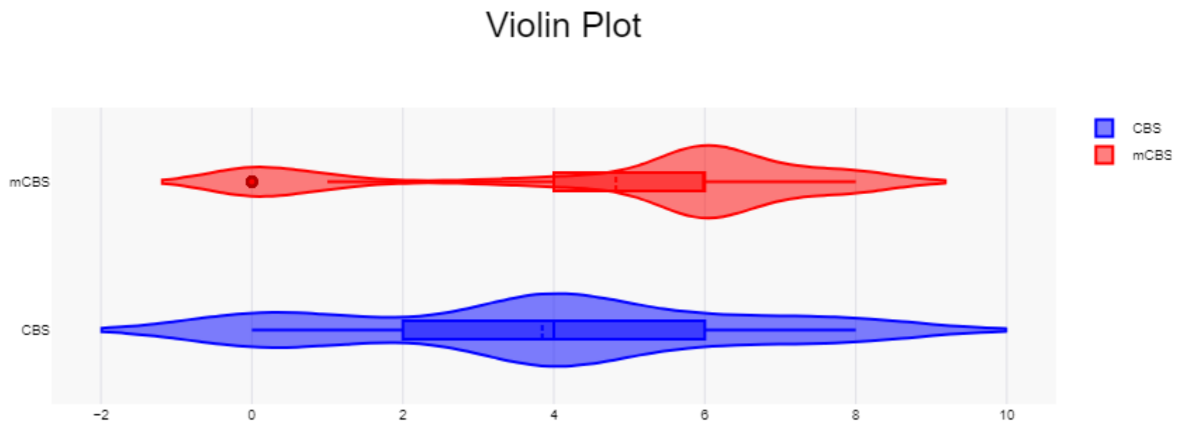

Chart 2. Violin plot of mCBS and CBS.

Scoring System	Average ± SD	P-value	Interpretation	Skewness	Median	IQR [Q3-Q1]
**NIHss**	14.83 ± 3.74	0.0002	Not Normal	Sym	15	18 - 12
**ASPECT**	5.56 ± 2.28	< 0.0001	Not Normal	Asym Left	6	7 - 4
**MRS 0**	0.44 ± 0.71	< 0.0001	Not Normal	Asym Right	0	1 - 0
**MRS 90**	2.81 ± 2.3	< 0.0001	Not Normal	Sym	2	5 - 1
**CBS**	3.84 ± 2.48	< 0.0001	Not Normal	Sym	4	6 - 2
**mCBS**	4.82 ± 2.69	< 0.0001	Not Normal	Asym Left	6	6 - 3.75

Table 1. Displaying the average values and inter-quartile range of the various scoring systems, along with distribution of normality and skewness of the data. Sym: Symmetrical, Asym: Asymmetrical, IQR: Inter-Quartile Range.

3-2. Correlations and Significance

The four previously established scoring systems were weighed against CBS and mCBS using Spearman's rank correlation, and results showed that almost all of them were significantly correlated, with the exception of MRS0 which had a borderline P-value against CBS (Table 2). Moreover, Results of the Spearman correlation indicated that there is a significant large positive relationship between CBS and mCBS, (rho = 0.892, p < 0.0001).

Scoring	Against CBS		Against mCBS	
	CC	P-value	CC	P-value
NIHss	-0.496	< 0.0001	-0.5693	< 0.0001
ASPECT	0.3618	0.00002	0.4058	< 0.0001
MRS 0	-0.1646	0.061	-0.2325	0.0077
MRS 90	-0.4486	< 0.0001	-0.5678	< 0.0001

Table 2. Showing the results of the Spearman's rank correlation test against CBS and mCBS. CC: Correlation Coefficient.

3-3. Bland and Altman Plot

The results of the Bland and Altman plot are illustrated in Figures 1 to 3. For CBS, due to the fact that the slope difference has a 95%CI that does not include zero when plotting with NIHss [0.98, 2.05] and MRS0 [-2.09, -1.68], a regression based limits of agreement was used. This was the case for all four systems when plotting with mCBS; NIHss [0.75, 1.9], MRS0 [-2.17, -1.78], MRS90 [-1.56, -0.23], and ASPECT [-0.43, -0.01]. Similarly, a regression method was used in plotting mCBS-CBS [-0.16, -0.01]. The results also show that NIHss and MSR90 had the best fit with CBS and mCBS, in line with the results of the Spearman's rank correlation.

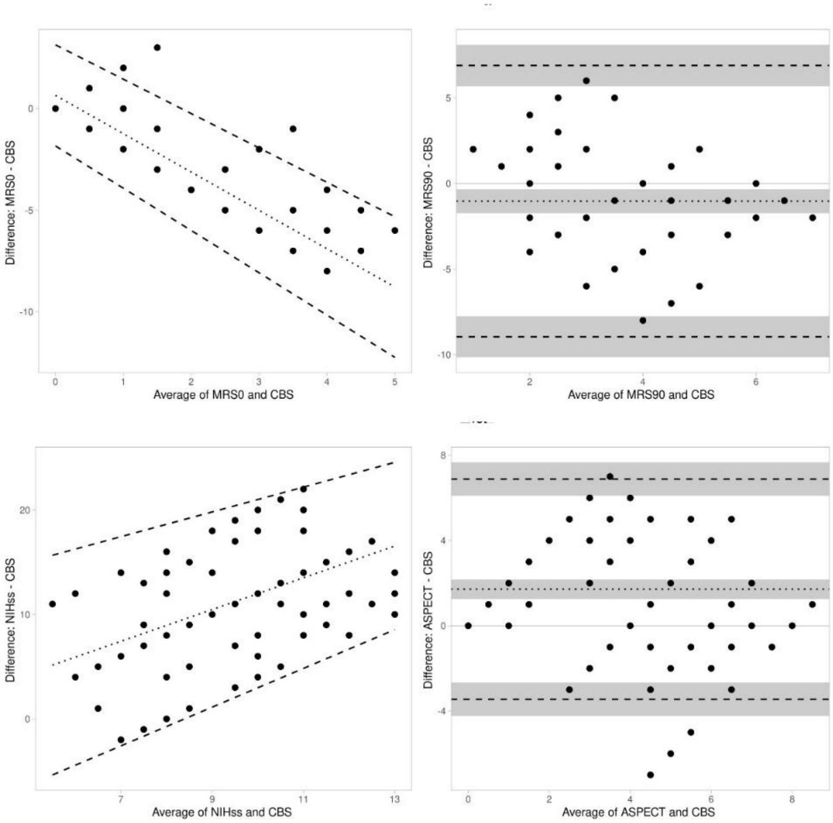

3

Figure 1. Showing the results of the Bland & Altman plot of CBS and the other four systems.

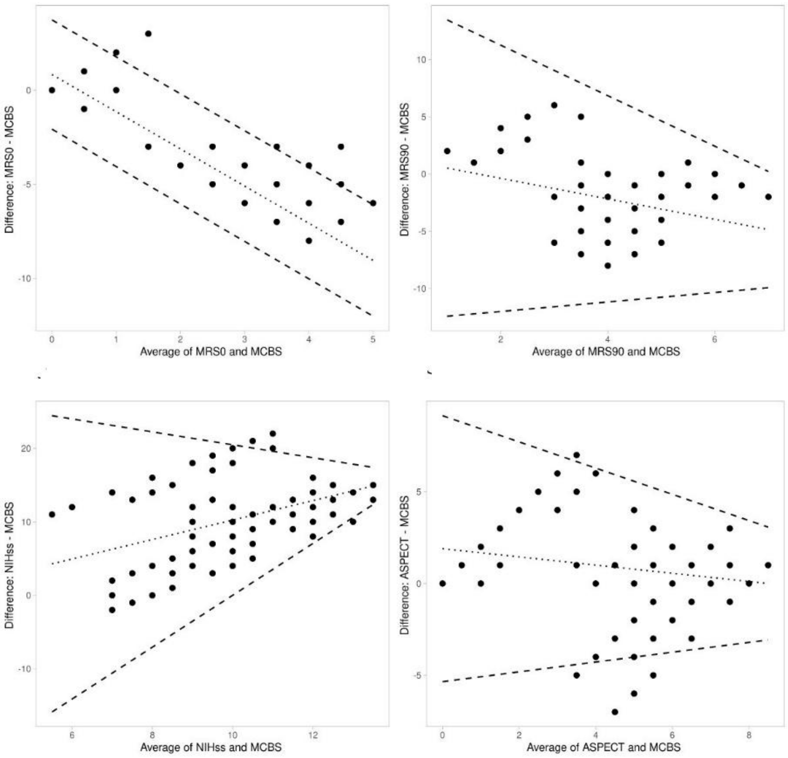

Figure 2. Showing the results of the Bland & Altman plot of mCBS and the other four systems.

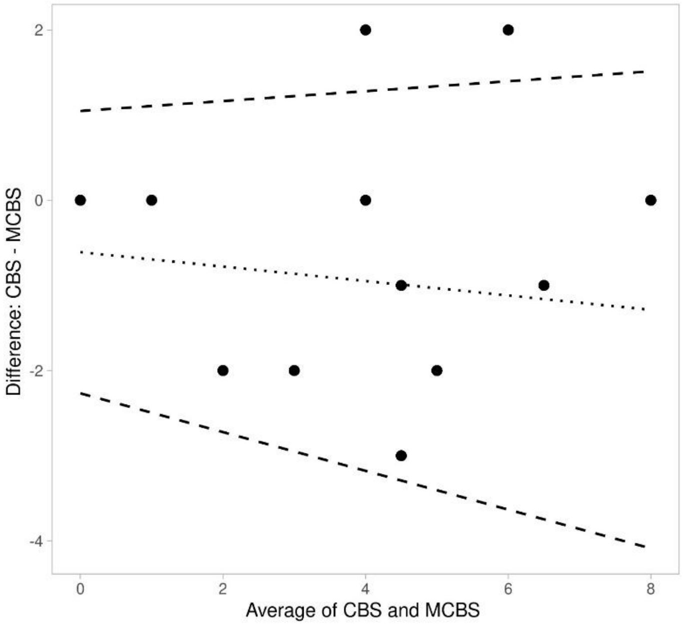

Figure 3. Showing the results of the Bland & Altman plot of mCBS and CBS.

3-4. Mortality, Disability, and Severity Correlation

In this study 29 patients died by the 90-day mark (MRS90 of 6), 83 had varying degrees of disabilities (MRS90 ≥ 2), and 70 were classified as severe (NIHss ≥ 15). Statistical difference was not seen amongst men and women in regards with these parameters using the Chi-squared test (Table 3). Additionally, both CBS and mCBS were significantly correlated with all three, although mCBS generally performed better (Table 4).

Parameter	N (%) in men	Chi-Squared	P-value
Mortality	19 (27.5%)	2.3	0.129
Disability	46 (66.7%)	0.503	0.4782
Severity	34 (49.3%)	1.227	0.2681

Table 3. Differences between mortality, disability, and severity amongst men and women.

Parameter	CBS		mCBS	
	Rho	P-value	Rho	P-value
Mortality	-0.2734	0.0016	-0.3126	0.0002
Disability	-0.411	<0.0001	-0.532	<0.0001
Severity	-0.399	<0.0001	-0.5	<0.0001

Table 4. Correlation between CBS and mCBS and mortality, disability, and severity. Significant correlations using Spearman's rank test.

3-5. ROC and AUC for Mortality

To further assess the predictive value of mCBS and CBS for mortality ROC curves and AUC were calculated (Charts 3 and 4). For CBS, the AUC was calculated as 0.681 with a P-value of 0.0045 (95%CI [0.593, 0.760]). Additionally, CBS had a Youden index J of 0.4032, with the associated criterion "≤1" having a sensitivity and specificity of 55.17% and 85.15%, respectively. mCBS comparatively performed better, with an AUC of 0.708 and a P-value of 0.0005 (95%CI [0.622, 0.784]). However, the Youden index J (0.4131), sensitivity (55.17%), and specificity (86.14%) were nearly identical. However, pairwise comparison of ROC curves showed no statistical significance (P-value = 0.3821).

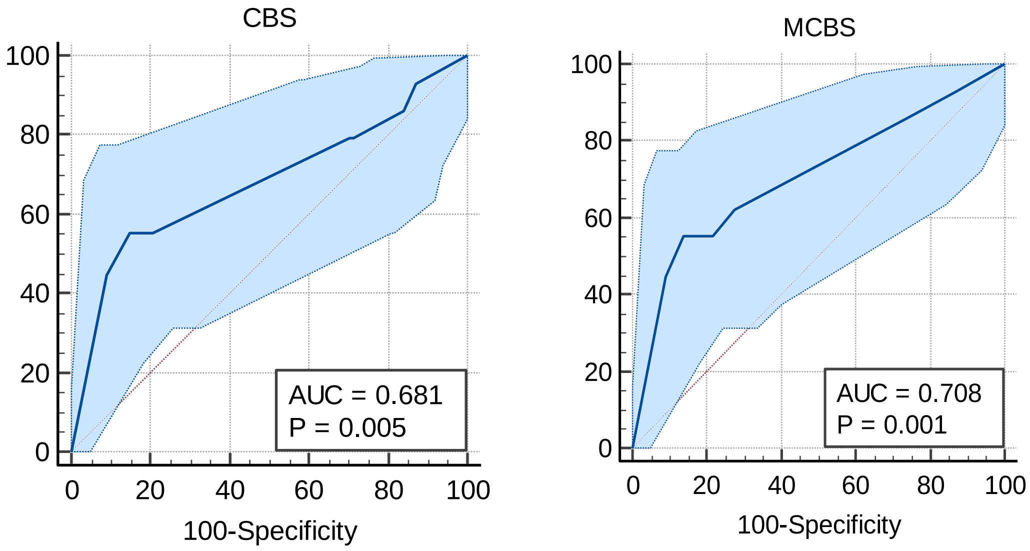

Charts 1 and 2. The mortality ROC curve for CBS and mCBS.

3-6. ROC and AUC for Disability

To assess the predictive value of CBS and mCBS for disability the patients were categorized into two groups, those without disability at the 90-day mark (MRS90 0-1) and those with disabilities (MRS90 2-6). For CBS, the AUC was calculated as 0. 735 with a P-value of <0.0001 (95%CI [0.651, 0.809]). The Youden J index was calculated at 0.4337, with the criterion "≤2" having a sensitivity and specificity of 43.37% and 100%. The AUC for mCBS was slightly higher at 0.807 (Chart 5), with a similar P-value of <0.0001 (95%CI [0.728, 0.871]). The J index was also higher at 0.5542 (Chart 6), however this time the criterion "≤5" had a specificity of 100% and a sensitivity of 55.42%. This time, unlike with mortality, pairwise comparison of the ROC curves was deemed statistically significant with a P-value 0.0019.

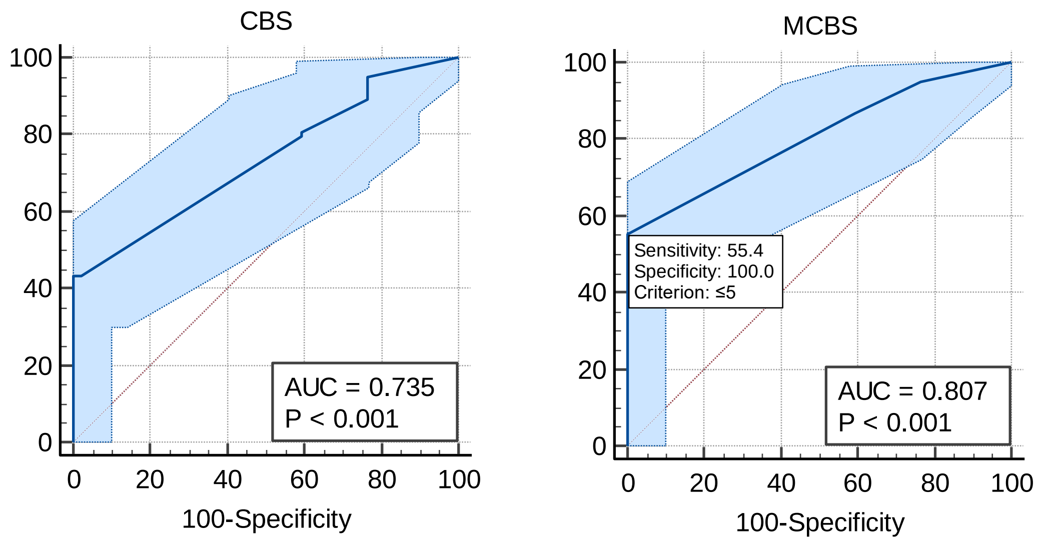

Charts 5 and 6. The disability ROC curve for CBS and mCBS.

3-7. ROC and AUC for Severity

To assess the predictive value of CBS and mCBS for severity the patients were categorized into two groups, severe stroke was rated as an NIHss score of 15 or higher. For CBS, the AUC was calculated as 0.72 with a P-value of <0.0001 (95%CI [0.635, 0.795]). The Youden J index was calculated at 0.3595, with the criterion "≤2" having a sensitivity and specificity of 44.29% and 91.67%. The AUC for mCBS was slightly higher at 0.778 (Chart 7), with a similar P-value of <0.0001 (95%CI [0.696, 0.846]). The J index was also higher at 0.4714 (Chart 8), however this time the criterion "≤5" had a specificity of 90% and a sensitivity of 57.14%. Similar to that of disability, pairwise comparison of the ROC curves was deemed statistically significant with a P-value 0.0156.

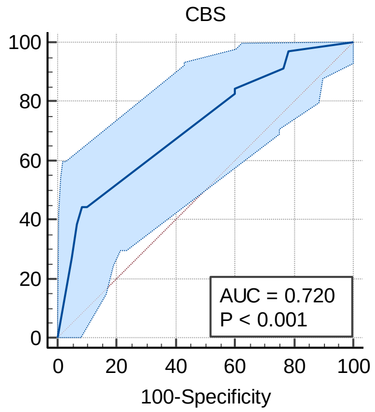

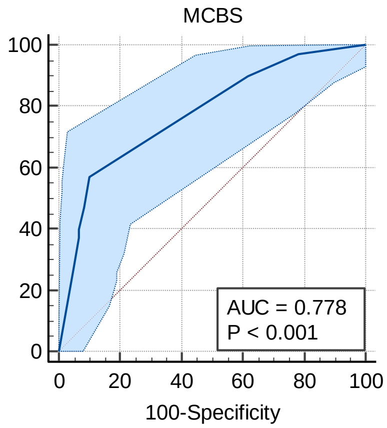

Charts 7 and 8. The ROC curve for CBS and mCBS.

3-8. Treatments and Outcomes

In our study, 54 patients received APT, 20 received TPA, and 53 received EVT. Differences in disability and severity were varied across all treatment modalities, although only patients that received APT had a significant reduction in mortality rates (Table 5). As seen in table 6, mCBS was better correlated to outcomes within the different treatment groups, particularly with mortality and severity in those who received TPA, and disability in EVT patients. The patients were further categorized according to MRS90 score as seen in table 7.

Parameter	P-value APT (n of 57)	P-value TPA (n of 20)	P-value EVT (n of 53)
Mortality	0.0349 (17)	0.3953 (3)	0.228 (9)
Disability	0.0055 (44)	0.3725 (11)	0.0307 (28)
Severity	0.0015 (40)	0.0205 (6)	0.1055 (24)

Table 5. Chi-squared test results of treatments and outcomes.

Parameter		CBS		mCBS	
		Rho	P-value	Rho	Rho
APT	Mortality	-0.369	0.0048	-0.387	0.0029
	Disability	-0.558	<0.0001	-0.613	<0.0001
	Severity	-0.378	0.0037	-0.387	0.0029
TPA	Mortality	-0.347	0.1335	-0.641	0.0023
	Disability	-0.470	0.0366	-0.662	0.0015
	Severity	-0.364	0.1143	-0.468	0.0373
EVT	Mortality	-0.0524	0.7095	-0.0250	0.8587
	Disability	-0.166	0.2356	-0.281	0.0414
	Severity	-0.405	0.0027	-0.510	0.0001

Table 6. Spearman's rank test results.

CBS	MRS90 ≤ 2	MRS90 > 2	mCBS	MRS90 ≤ 2	MRS90 > 2
**0**	0	21	**0**	1	21
**1**	1	8	**1**	1	7
**2**	3	2	**2**	0	0
**3**	1	0	**3**	0	2
**4**	37	20	**4**	4	2
**5**	0	1	**5**	1	7
**6**	10	5	**6**	36	18
**7**	0	5	**7**	10	5
**8**	13	2	**8**	13	2

Table 7. Counts and categorization of patients according to MRS90 scores.
