# Supplementary material for: Prognostic value of the modified clot burden score in predicting outcomes of acute ischemic stroke patients
Source: BMC Neurol. 2026 Feb 2;26:139. doi: 10.1186/s12883-026-04626-w (PMC12955203; doi:10.1186/s12883-026-04626-w)
Supplement: Supplementary file 2 — Supplementary Material 2. [file 12883_2026_4626_MOESM2_ESM.docx]

**Supplementary Table 1.** Chi-squared test results of treatments and outcomes.

| Parameter | P-value APT (n of 57) | P-value TPA (n of 20) | P-value EVT (n of 53) |
| --- | --- | --- | --- |
| Mortality | 0.0349 (17) | 0.3953 (3) | 0.228 (9) |
| Disability | 0.0055 (44) | 0.3725 (11) | 0.0307 (28) |
| Severity | 0.0015 (40) | 0.0205 (6) | 0.1055 (24) |

**Supplementary Table 2.** Chi-squared test results of treatments and outcomes.

| Variable | CBS (without sex) | CBS (with sex) | mCBS (without sex) | mCBS (with sex) |
| --- | --- | --- | --- | --- |
| Age | 0.552 | 0.894 | 0.505 | 0.812 |
| ASPECT | 0.254 | 0.311 | 0.302 | 0.34 |
| NIHss | 0.823 | 0.811 | 0.904 | 0.87 |
| Treatment | 0.93 | 0.865 | 0.759 | 0.976 |
| Sex | - | 0.12 | - | 0.18 |
| Overall | 0.538 | 0.339 | 0.724 | 0.559 |

**Supplementary Table 3.** Results of the ordinal logistic regression.

| Variable | CBS (without sex) | CBS (with sex) | mCBS (without sex) | mCBS (with sex) |
| --- | --- | --- | --- | --- |
| Age | 0.696 | 0.657 | 0.672 | 0.592 |
| ASPECT | 0.037 | 0.033 | 0.332 | 0.246 |
| NIHss | 0.001 | 0.001 | 0.0001 | 0.0001 |
| Treatment | 0.667 | 0.677 | 0.315 | 0.321 |
| Sex | - | 0.685 | - | 0.258 |
| Overall | < 0.0001 | < 0.0001 | < 0.0001 | < 0.0001 |

**Supplementary Table 4.** Results of the binary logistic regression.

| **Parameter** | **P-value APT (n of 57)** | **P-value TPA (n of 20)** | **P-value EVT (n of 53)** |
| --- | --- | --- | --- |
| **Mortality** | 0.0349 (17) | 0.3953 (3) | 0.228 (9) |
| **Disability** | 0.0055 (44) | 0.3725 (11) | 0.0307 (28) |
| **Severity** | 0.0015 (40) | 0.0205 (6) | 0.1055 (24) |
